# Notes and News

**Published:** 1898-10-22

**Authors:** 


### Notes and News.

(Collected and Communicated.)

The post of medical superintendent of the disinfecting service at Suez and Moses Wells is vacant. The commencing salary is £316 10s. per annum, with a house. Preference will be given to candidates having theoretical and practical knowledge of bacteriology and epidermology. The post is under the Conseil Sanitaire Maritime et Quarantenaire of Egypt, and applications should be sent on or before January 6th to the president, Dr. Ruffer, Alexandria, Egypt.

Complaints reach us of the delays in the dispensing department of the Civil Hospital at Colombo. So far the Government have only sanctioned the employment of one qualified dispenser, and as he has the charge of the medical stores also, he is frequently called away from the dispensing of prescriptions, which sometimes number 200 a day. Dr. Garvin, physician to the hospital, urgently appeals to the Government for further assistance in the dispensary.

The lot of the lady guardian is not unmixed with unpleasantness at Rochdale. Here three lady guardians were appointed, and, according to the custom when their male confederates were elected, they were required "to pay their footing," the proceeds being devoted to "a drink fund." The matter found its way into the local papers, where a former chairman of the board expressed himself highly indignant at the objections raised to the custom as applying either to male or female guardians. He explained that if ladies would be guardians, and enter public offices, they must take all responsibilities.

The following gentlemen have been elected officers of the British Laryngological, Rhinological, and Otological Association for the ensuing year 1898-99: President—Dr. Middlemas Hunt (Liverpool); vice-presidents—Dr. Davison (Bournemouth), Mr. John Bark (Liverpool), and Mr. Wyatt Wingrave; council—Dr. Dundas Grant, Dr. W. Hill, Dr. Greville MacDonald, Dr. Abercrombie, Dr. Percy Jakins, Dr. H. Woods (Dublin), and Dr. A. B. Kelly (Glasgow); treasurer—Dr. McNeil Whistler; hon. secretaries—Mr. St. George Reid and Dr. Furniss Potter.

Although the plague shows continued abatement in Bombay city, in other parts of the presidency the epidemic is still extending. In the Baroda and Mysore States there is an increase; also in Hyderabad and some districts of the Madras presidency.

The Cardiff Infirmary is to receive a most welcome and generous gift in the form of a new operating theatre from Mr. Thomas Webb. The building, which is to cost £1,000, will be erected as a memorial to the late Mrs. Webb. Mr. Webb reserves to himself the right of selecting the architect.

It must be gratifying to the Hospital Saturday Fund to find that their public-spirited abandonment of the street collection has been so practically recognised by many friends. Not a few business firms adopted special methods of securing contributions for them on Hospital Saturday. In one case a dummy figure of a boy, with bandaged head and arm, was placed at the entrance of a business house to graphically plead for real sufferers. The ladies have met with a good deal of success with the collecting cards and subscription lists with which they were supplied.

The conscience clause in the Vaccination Act has evidently not raised a feeling of admiration in France, and there is evidence to show that public opinion is steadily setting in the direction of compulsory vaccination at the very moment when our Government has abandoned it. At a recent meeting of the Department of Hygiene of the French Society for the Advancement of Science, a motion was brought forward recommending compulsory vaccination for all the native and foreign population at Tunis, in view of the prevalence of small-pox amongst the unvaccinated portion of the community.

Dr. Rufenacht Walters says that there are at the present time nearly a hundred sanatoria for consumptives in existence, being built, or projected. In Germany alone there are now 43 in working order, of which 17 are for patients paying from £3 to £6 per week. The German sickness insurance companies have this year set aside between three and four million marks for the erection or subvention of sanatoria for consumptives. In America there are 19, besides one which is being built. These institutions are intended for presumably curable patients. Advanced and incurable cases should be sent to special homes or refuges.

At the Hospital for Consumption and Diseases of the Chest, Brompton, the following lectures and demonstrations will take place on Wednesdays, at four p.m., commencing October 26th: On "Bronchiectasis," by Dr. Habershon; "Anæmic Murmurs," by Dr. Maguire; "Cases Illustrating Types of Tuberculosis, with special reference to Diagnosis and Prognosis," by Dr. Acland; "The Symptomatic Treatment of Pulmonary Tuberculosis," by Dr. Green; "Asthma and its Treatment," by Dr. S. Martin, F.R.S.; "Principles of Treatment of Pulmonary Tuberculosis," by Dr. Biss; "Pulmonary Abscess," by Mr. Godlee; and on "Hæmoptysis," by Dr. Schorstein, on December 14th, concluding the series. The lectures are free to all qualified medical practitioners, and to students attending the practice of the hospital.

